# Experiences of young adults with cardiovascular disease in using digital health: a protocol for a qualitative systematic review and meta-synthesis from the perspective of media affordances

**DOI:** 10.3389/fcvm.2026.1806112

**Published:** 2026-05-22

**Authors:** Yi Cen, Ruolin Qiu, Ying Liu, Yuexiao Zheng, Zeyi Zhang, Yifan Zhu, Zhendong Lan

**Affiliations:** 1 School of Public Health and Nursing, Hangzhou Normal University, Zhejiang, Hangzhou, China; 2 Zhejiang Philosophy and Social Science Laboratory for Research in Early Development and Childcare, Hangzhou Normal University, Zhejiang, Hangzhou, China

**Keywords:** cardiovascular diseases, digital health, meta-synthesis, systematic review, young adults

## Abstract

**Introduction:**

Cardiovascular disease (CVD) is a leading cause of disability and mortality worldwide, with a rising prevalence among young adults. Digital health technologies (DHTs) offer potential support for disease management, yet their integration into the daily lives of young adults with CVD remains poorly understood. This study aims to systematically review and synthesize qualitative evidence on how young adults with CVD experience and engage with DHTs, from the perspective of media affordances.

**Methods:**

This review will employ a qualitative systematic review and meta-synthesis. We will search seven databases (Cochrane Library, PubMed, Web of Science, Scopus, Embase, CINAHL, PsycINFO) for qualitative and mixed-methods studies published from January 2013 to August 2025. Studies involving young adults (aged 18–49) with CVD who have used DHTs for disease management will be included. Two independent reviewers will screen studies, assess methodological quality, extract data, and synthesize findings, using the JBI Critical Appraisal Checklist for Qualitative Research for quality assessment and thematic synthesis guided by the Theory of Media Affordances for data synthesis. Confidence in the synthesized findings will be assessed using the GRADE-CERQual approach.

**Clinical Trial Registration:** https://www.crd.york.ac.uk/PROSPERO/view/CRD420251138307, PROSPERO registration number CRD420251138307

## Introduction

Cardiovascular disease (CVD) is globally recognized as a principal cause of disability and mortality, with a notable trend toward younger onset (1–4). According to the Global Burden of Disease (GBD) study, the global prevalence of CVD was estimated at 523 million cases in 2019, resulting in 18.6 million deaths, of which approximately 1.2 million occurred in individuals under 50 years of age. Consequently, CVD has been identified as a major contributor to premature mortality in young adults (5, 6). This public health challenge is closely linked to rapid socioeconomic development and shifting lifestyle patterns (1), indicating that a substantial number of individuals are diagnosed with chronic conditions during their most productive life years, thereby confronting distinct healthcare and lifestyle challenges.

Young adulthood is a critical period for educational advancement, career establishment, and family formation. A CVD diagnosis during this phase requires patients balance intensive disease management with the pursuit of fundamental life objectives (7, 8), which substantially compromises quality of life and frequently precipitates psychological comorbidities including anxiety and depression (9). Researches have demonstrated that younger populations experience considerable socioeconomic burdens (10) and exhibit greater vulnerability to psychosocial challenges compared to the older adults (11). Several primary self-management challenges have been identified among young adult CVD patients. First, CVD typically presents with an insidious onset and non-specific early symptoms, often resulting in diagnostic delays or insufficient recognition of disease severity (12). Second, the protracted disease course frequently leads to complacent attitudes and diminished motivation for sustained self-management (13). Third, significant limitations persist within existing healthcare support systems. Although community-based management models are progressively developed, their service orientation remains predominantly focused on older adults and common chronic conditions such as hypertension and diabetes. These management approaches typically emphasize standardized guidance, while providing less personalized support that addresses the technological preferences, psychosocial requirements, and lifestyle diversity specific to young adult CVD patients, thereby failing to deliver patient-centered, comprehensive, and adaptive care (14–16). Furthermore, with accelerating population aging, contemporary young adult CVD patients are increasingly likely to experience premature transitions into a period characterized by multimorbidity, potentially generating dual pressures on national healthcare and social security systems (15). Consequently, innovative support strategies that align with this population's lifestyles, values, and technology utilization patterns must be urgently developed to enhance self-management efficacy and establish proactive measures for mitigating future public health burdens. Although definitions of “young adulthood” vary across studies, there is growing recognition that the period up to age 50 represents a continuous transitional phase in terms of disease onset, psychosocial development, and health behavior formation, particularly in the context of chronic cardiovascular conditions (17, 18). Therefore, to comprehensively capture the experiences of this population, this review adopts an expanded age range of 18 to 49 years.

In the Global Strategy on Digital Health, the World Health Organization (WHO) defined digital health technologies (DHTs) as the use of digital technologies to improve health-related knowledge and practices, encompassing mobile applications, electronic health records, telehealth platforms, wearable devices, artificial intelligence (AI), and virtual reality technologies (19). DHTs have been increasingly integrated into healthcare delivery, serving critical functions in disease surveillance, risk prediction, health management, and care continuity (20). Recent research has documented the multifaceted benefits of DHTs in CVD management, with a growing body of evidence focusing specifically on young and middle-aged populations. A systematic review by Niu and Li, which included 12 randomized controlled trials involving 1,879 participants aged 18–59 years, demonstrated that digital health interventions significantly improved blood pressure control and enhanced medication adherence in young and middle-aged hypertension patients (21). An intervention study targeting young adults aged 18–35 years showed that a telehealth-based health program effectively improved cardiovascular risk-related behaviors, such as dietary fat intake, with participants reporting predominantly positive experiences (22). Furthermore, a discrete choice experiment examining mobile app feature preferences among individuals with chronic heart disease (mean age 50.5 years) found that when app features aligned with user preferences—such as monitoring feedback, personalized health education, and flexible symptom diaries—user adoption rates increased from 84% to 92% (23). Thus, through smartphone applications, wearable devices, and telehealth platforms, DHTs offer novel approaches for self-monitoring, personalized feedback, and health information access, enabling informed decision-making while creating innovative solutions for earlier chronic disease prevention, diagnosis, and management (24).

For individuals born around 1990, young adulthood coincides with digital nativity, positioning this cohort ideally positioned to adopt and benefit from DHTs (25). However, a substantial disparity between technological usability and practical effectiveness has been documented, suggesting that adequate usability alone cannot ensure sustained user engagement or clinical efficacy (26–28). The ultimate effectiveness of technology is determined by its capacity to integrate into daily routines, fulfillment of individualized needs, and provide continuous value. Numerous DHT implementations are challenged by high user churn rates and limited long-term user engagement, primarily attributable to intervention designs that inadequately address user experience, how technologies are perceived, interacted with, and adopted (29–32). Several systematic reviews have examined the effectiveness and implementation of DHTs in cardiac rehabilitation contexts (31), and other investigations have addressed the cost-effectiveness of digital health interventions in CVD management (33). However, these studies have predominantly emphasized clinical effectiveness outcomes or collective experiences across broad age groups, failing to address the distinctive adoption barriers, facilitating factors, and utilization experiences specific to young adults with CVD.

To move beyond a superficial descriptions of DHTs experiences among young adults with CVD, this study introduces the Theory of Media Affordances—a framework proposed by the Chinese scholar Pan in 2017—as its core analytical framework (34), providing theoretical support for systematically analyzing how DHTs characteristics shape user experience. The concept of affordance originated in ecological psychology (35), initially referring to the possibilities for action that an environment provides to an animal. This theory extends the concept, positing that technologies are not neutral tools but rather provide users with a range of action possibilities, namely affordance, through their functional features. These possibilities must be perceived and actualized by users to realize their value (34). The theory comprises three principal dimensions: production affordances, social affordances, and mobile affordances. In this study, production affordances refer to how technology enables users to perform specific health behaviors. This is supported by Zhang, who found that the functions of chronic disease management apps, such as health data recording, task reminders, and personalized feedback, were key factors influencing users' continuous usage intentions (36). Social affordances in this study emphasize how technology mediates or transforms communication and connections between patients and healthcare providers, family members, and peers. In discussing adolescent health services, O'Malley indicated that digital technologies could facilitate private communication, peer support network building, and patient-provider interaction, which were considered crucial for meeting the unique psychosocial needs of this age group (37). Smith supported this point in their study on childhood chronic lung disease management, in which the information sharing and remote collaboration functions of digital tools were identified as core factors influencing adoption by healthcare providers and parents (38). Mobile affordances refer to how technological interfaces and information architectures influence users' perception, understanding, and operational flow. The early work by Klasnja and Pratt emphasized that the user experience of mobile health products was rooted in their interface design and information architecture, which collectively determined whether the technology could be seamlessly integrated into users' daily lives (39). Furthermore, each dimension encompasses distinct characteristics. The three key dimensions of the Theory of Media Affordances and their definitions in this study are detailed in Table 1. Although an increasing number of publications examined individual dimension of media affordances, and quite a few researches have been conducted in healthcare fields and chronic disease populations, studies systematically applying the media affordance perspective to cardiovascular populations, particularly young adults, remain scarce, let alone their experiences of using DHTs. The selection of the Theory of Media Affordances as the core analytical framework is grounded in its distinct advantages over alternative theoretical perspectives. Traditional technology acceptance models, such as the Technology Acceptance Model (TAM) (40) and the Unified Theory of Acceptance and Use of Technology (UTAUT) (41), primarily focus on users' attitudes, perceived usefulness, and behavioral intentions, often treating technology as a neutral tool whose effects are mediated by individual perceptions. These models have been widely applied in digital health research, with systematic reviews documenting their use in examining patient and provider adoption of health technologies (42, 43). In contrast, the Theory of Media Affordances emphasizes the relational and dynamic nature of technology use—specifically, how technological features enable or constrain actions within specific contexts (34, 35). This relational perspective is particularly suited for understanding how young adults, who are typically digital natives, actively engage with DHTs in their daily lives, as it captures the reciprocal shaping between technological materiality and user practices (25, 44).

**Table 1:** Key dimensions and characteristics of theory of media affordances.

Dimensions	Characteristics	Definitions in this study
Production Affordances	Edit-ability; Review-ability; Replicability; Scalability; Associability	How technology enables users to perform specific health behaviors.
Social Affordances	Greet-ability; Emotion-ability; Coordinate-ability; Connect-ability	How technology mediates or transforms communication and connections between patients and healthcare providers, family members, and peers.
Mobile Affordances	Portability; Availability; Locatability; Multimediality	How technological interfaces and information architectures influence users’ perception, understanding, and operational flow.

Therefore, to enhance the understanding of the aforementioned issues, a rigorous qualitative systematic review and meta-synthesis will be conducted. This study will systematically retrieve, appraise, and synthesize existing qualitative researches on the experiences of young adult with CVD using DHTs. The primary objective is to explain, through the systematic synthesis of available evidence, how the different affordances of DHTs influence the disease management practices and experiences of this population. To achieve this objective, the following core research questions will be addressed: (1) How do young adults with CVD perceive and actualize the production, social, and mobile affordances of DHTs in their disease management? (2) How do these affordances interact with each other and with individual, social, and contextual factors to shape their engagement and experiences? (3) What barriers and facilitators influence the integration of DHT affordances into their daily lives?

## Methods

### Study design and registration

Meta-synthesis is recognized as an analytical approach in qualitative systematic reviews that not only integrates fragmented evidence and reveals underlying patterns but also systematically addresses research questions. Through the systematic integration of findings from multiple qualitative studies, summarized themes (45) will be generated to achieve a in-depth interpretation of the experiences of young adults with CVD using DHTs. The study protocol was developed in accordance with the PRISMA-P (Preferred Reporting Items for Systematic Review and Meta-Analysis Protocols) 2015 statement (46) and has been prospectively registered with the International Prospective Register of Systematic Reviews (PROSPERO) (registration number: CRD420251138307). The entire research process will adhere to the Enhancing Transparency in Reporting the Synthesis of Qualitative Research (ENTREQ) statement, ensuring transparency and reliability of the findings. The study period is scheduled be conducted from September 2025 to September 2026. Any protocol amendments during this period will be recorded in the PROSPERO registry and explained in the final report.

### Search strategy

A comprehensive three-stage search strategy will be developed and implemented (47). Search terms will be formulated based on the SPIDER framework (Sample, Phenomenon of Interest, Design, Evaluation, Research Type) (48). This framework is specifically designed for qualitative and mixed-methods research. Compared with traditional PICO/PICOS frameworks, it ensures comprehensive coverage of terms related to the study population, core phenomena, and research design by focusing on research design and type (49). The three stages of this study are as follows: (1) Preliminary Search: Initial searches will be conducted in the PubMed and CINAHL databases to identify relevant keywords and Medical Subject Headings (MeSH) terms based on the SPIDER framework (Table 2) and to refine the final search strategy. (2) Systematic Search: A formal systematic search will be performed in Cochrane Library, PubMed, Web of Science, Scopus, Embase (via Ovid), CINAHL, and PsycINFO for all relevant literature published from January 1, 2013, to August 31, 2025. This time period was selected to capture studies conducted after the widespread adoption of smartphones in 2012. (3) Reference Screening: The reference lists of all included studies and relevant review articles will be manually searched to identify additional potentially eligible studies.

**Table 2:** Search terms.

SPIDER elements	Search terms
S-Sample	Young Adult(MeSH); Emerging adult*; Youth*
PI-Phenomenon of Interest	MeSH: Cardiovascular Diseases; Heart Diseases; Hypertension; Heart Failure; Myocardial Infarction; Coronary Artery Disease; Cardiomyopathies Free text: Cardiac Event*; Cardiac Disorder*; High Blood Pressure; Cardiac Failure; Heart Attack; Arrhythmia; Dysrhythmia; Coronary Atheroscleros*; Heart Defect*; Myocardial Disease
	MeSH: Digital health; Telemedicine; Digital Technology; Mobile Applications; Wearable Electronic Devices Free text: Virtual Medicine; Mobile Health; mHealth; Telehealth; eHealth; Telecare; Digital Electronic*; Mobile Phone*; Application*; Software*; Smartphone*; Wearable
D-Design	MeSH: Case Reports; Grounded Theory; Interview; Focus Groups; Qualitative Research Free text: Mix method*; Case study; Grounded Theory; Narrat*; Field study; Content analysis; Thematic analysis; Phenomen*; Qualitative study
E-Evaluation	experienc*; Satisfact*; Attitude*; Opinion*; Percep*; Process*; Feel*; View*; Stor*; Emotion*
R-Research Type	Qualitative Research(MeSH); Mix method*; Qualitative study

The search strategy will integrate relevant terms based on the SPIDER framework. The full search strategy for PubMed is provided in Supplementary Appendix S1.

### Study selection and eligibility criteria

#### Selection process

Search results will be imported into EndNote 2025 (Clarivate Analytics, USA) for deduplication. Subsequently, two reviewers (Y.C, Y.Z) will independently screen the titles, abstracts and full texts using a shared Microsoft Word document. Any disagreements will be resolved through discussion or by consulting a third reviewer (R.Q). The study selection process will be documented using a PRISMA flow diagram.

#### Eligibility criteria

The inclusion criteria for this study are formulated based on the SPIDER framework, which is also suitable for searching and screening qualitative and mixed-methods studies (49). This study will focus specifically on young adult with CVD aged between 18 and 49 years. This age range (18–49 years) reflects the extended transitional period from early adulthood to midlife, during which CVD onset, self-management needs, and digital health engagement patterns share considerable commonalities. Expanding the upper age limit to 49 ensures a more comprehensive capture of young adult experiences with CVD. This includes congenital heart disease (CHD), as individuals with CHD represent a significant and growing subgroup of young adults with cardiovascular conditions. For mixed-methods studies, only the qualitative components (e.g., interview data, qualitative findings, participant quotations) will be included for synthesis; quantitative data will not be systematically extracted or synthesized, though they may be referenced to provide contextual understanding of the study setting or participant characteristics. The specific inclusion and exclusion criteria are detailed in Table 3 and Table 4, respectively. Studies for which the full text cannot be accessed or from which data cannot be extracted will also be excluded.

**Table 3:** Inclusion criteria.

SPIDER element	Inclusion criteria
S (Sample)	Young adults aged 18–49 years. Clinically diagnosed with any CVD (including congenital heart disease). Time since diagnosis is over 6 months.
PI (Phenomenon of Interest)	Use of any DHTs for CVD management within the past year.
D (Design)	Qualitative studies (e.g., phenomenology, the grounded theory, ethnography) or the qualitative component of mixed-methods studies, employing methods such as content analysis, thematic analysis, etc.
E (Evaluation)	User engagement, perceived benefits, barriers to use, emotional responses, self-management, social support, perceived facilitators or barriers, etc.
R (Research type)	Peer-reviewed English language qualitative studies.

**Table 4:** Exclusion criteria.

SPIDER element	Exclusion criteria
S (Sample)	– Individuals currently hospitalized for acute cardiovascular events (e.g., acute myocardial infarction, decompensated heart failure) or other critical illnesses.
	– Individuals with severe comorbidities (e.g., major mental illnesses such as psychosis or dementia, significant cognitive impairment, terminal illness) that may severely affect their ability to participate in digital health interventions or articulate their experiences.
PI (Phenomenon of Interest)	Studies focusing solely on cardiovascular risk factors (e.g., obesity) rather than disease management.
D (Design)	Mixed-methods studies containing only quantitative research, systematic reviews, meta-analyses, commentaries, conference abstracts, study protocols, and editorials.

### Exclusion criteria

#### Quality appraisal

The methodological quality of the included studies will be independently assessed by two trained reviewers (Y.C, Y.Z). The primary aim of this appraisal is to assess the methodological rigor of each study and its potential impact on the credibility of the findings, rather than to simply exclude studies. The Joanna Briggs Institute (JBI) Critical Appraisal Checklist for Qualitative Research will be used for this process. The appraisal covers aspects such as the congruence between philosophical perspective and methodology, consistency between research methods and data collection/analysis, statement of the researcher's cultural or theoretical orientation, and whether appropriate ethical approval was obtained. Detailed results will be presented in a table. This tool places particular emphasis on assessing researcher reflexivity (50), which is crucial for evaluating the interpretive rigor of qualitative studies and can be directly linked to the subsequent ConQual (Confidence in the Evidence from Qualitative Research) assessment of confidence in the evidence.

#### Data extraction

Data extraction will be performed independently by two researchers (Y.C, Y.Z) using a standardized form adapted from the JBI Data Extraction Tool for Qualitative Evidence Synthesis. The form (Table 5) will cover study characteristics, participant characteristics, methodological characteristics and other relevant information. This aims to comprehensively capture the study context, methodological details, and core findings. Before extraction, the researchers will undergo training and perform pilot extraction on two to three studies to ensure consistent understanding and application of the form. Any discrepancies arising during the extraction process will be resolved through discussion and consensus; if necessary, a third researcher (R.Q) will be consulted to reach a decision. All extracted data will be consolidated into a single table in preparation for subsequent synthesis analysis.

**Table 5:** Data extraction content.

Category	Content to be extracted
Study Characteristics	First author, publication year, country Study aim Study design/methodology Theoretical framework
Participant Characteristics	Sample size Age Gender Type of cardiovascular disease DHT usage experience
Methodological Characteristics	Data collection methods Data analysis methods Statement of researcher reflexivity
Intervention and Context	Specific types of DHTs used Core functions Duration and frequency of use Context of study implementation.
Qualitative Findings	Themes, categories, and metaphors explicitly reported or interpreted by authors Extracted line by line from the findings section
Supporting Data	Illustrative quotes supporting each finding (with participant ID or source noted)
Credibility of Findings	Authors’ assessments of the credibility of their own findings.

#### Data synthesis

The data synthesis for this study will employ thematic synthesis, integrating both inductive and deductive reasoning (51). This method aims to generate new theoretical insights by systematically coding, summarizing, and interpretively integrating the findings from primary qualitative studies, thereby providing an in-depth explanation of how different affordances of DHTs influence the disease management practices and experiences of young adult with CVD. The synthesis process will be conducted independently by two researchers (Y.C, Y.Z) and managed using NVivo 15 software (QSR International, Australia) to enhance rigor and transparency. All disagreements will be resolved through consensus; if consensus cannot be reached, a third researcher (R.Q.) will be consulted for resolution. To account for heterogeneity in DHT interventions, we will categorize the included studies by DHT type (e.g., wearable devices, mobile applications, telehealth platforms) and explore whether patterns of affordance actualization differ across categories. The thematic synthesis involves the following three stages:

1. Inductive Stage-Line-by-Line Coding: Reviewers will read the authors’ findings, extracted from all included studies line by line, including themes, metaphors, and key statements from the primary studies. Initial codes will be generated through open coding of the text. This process emphasizes staying close to the raw data and avoiding premature influence from pre-existing theoretical frameworks. Coding will be performed using NVivo 20 (QSR International, Australia) to ensure systematic labeling and categorization of text segments.
2. Inductive Stage-Development of Descriptive Themes: Based on the initial codes, reviewers will iteratively compare and group codes with similar meanings to organize them into more general descriptive themes. These themes remain closely related to the content of the primary studies and aim to systematically present the shared experiences and barriers of young adult with CVD in using DHTs. The formation of descriptive themes will be visualized using thematic maps or tree diagrams to ensure clear logic and hierarchy.
3. Deductive Stage-Generation of Analytical Themes: Building upon the descriptive themes, reviewers will introduce the Theory of Media Affordances as an interpretive lens to guide the interpretive integration. This stage requires moving beyond the content of the primary studies by combining descriptive themes with the research questions to develop new theoretical insights or explanatory hypotheses. Analytical themes should directly address the study aims and provide actionable guidance for practice.

To ensure the rigor of the synthesis process, two reviewers (Y.C, Y.Z) will independently perform coding and theme development at each stage, reaching consensus through ongoing discussion. In case of disagreements, a third reviewer (R.Q) will be involved in the decision-making process. The entire process will be reviewed and approved by the research team to ensure the reliability of the results and the consistency of interpretations.

#### Assessing confidence in the findings

The GRADE-CERQual approach (52) will be used to assess confidence in each synthesized finding. This assessment system examines four core components: methodological limitations, relevance, coherence and adequacy of data. Methodological limitations refer to problems in the design or conduct of the primary studies contributing to the finding. Relevance concerns the extent to which the primary study evidence is applicable to the review question in terms of population, context, and intervention. Coherence reflects whether the finding presents a clear and compelling pattern across the data and provides a convincing explanation for any variations, including accounting for contradictory data. Adequacy of data refers to the richness and quantity of data supporting the finding. The overall confidence in each synthesized finding will be assessed as high, moderate, low, or very low.

## Discussion

Confronted with the dual core challenges of a long-term insidious disease course and the specific conflicts characteristic of their life stage, young adults with CVD are in urgent need of digital health solutions that are closely aligned with their circumstances. To gain an in-depth understanding of the complex experiences of this population in their interactions with technology, this protocol adopts the Theory of Media Affordances, a relatively comprehensive and emerging theoretical lens.

Among numerous potential theories, Communicative Theory represents a valuable perspective that shares similarities with the Theory of Media Affordances. Research has previously explored the patient-technology relationship from a communication perspective. For instance, Zhang, in a study on smartphone use among individuals with mental health conditions, analyzed how devices mediated patients' self-expression and external communication (53). However, Communicative Theory traditionally focuses on information transmission, persuasion processes, and interpersonal relationship building. When analyzing DHTs, it often directs researchers' attention to the communicative effects of the technology, such as information clarity, persuasiveness, and the quality of patient-provider communication (54). Critically, it has been argued that this perspective tends to treat technology as a neutral conduit, potentially underestimating the shaping influence of the materiality, functional architecture, and interactive logic of the technological platform itself on user behavior (55).

In contrast, the Theory of Media Affordances (34) adopted in this protocol shifts the analytical focus from what the technology communicates to what the technology itself enables users to do, that is, the possibilities for action it affords users. This shift provides a more explanatory framework for analyzing the user experience of DHTs and has been demonstrated to be crucial for understanding the adoption and sustained use of interactive technologies (44, 56, 57). Several distinct advantages are associated with its use as the core framework for this study.

First, the Theory of Media Affordances provides a powerful theoretical perspective for understanding the gap between the usability and effectiveness of DHTs. The failure of some DHTs to achieve intended outcomes is often not due to erroneous information content, but rather because their functional design fails to effectively embed into users’ daily life practices (58). By analyzing the production affordances and mobile affordances of technology, the Theory of Media Affordances offers precise conceptual tools for diagnosing and addressing this integration problem. Davis and Chouinard (59) noted that the actual influence of technological functions depends on their enforcement in specific contexts, namely, whether the technology encourages, allows, or precludes certain user behaviors, while also being constrained by user perception, operational ability, and sociocultural legitimacy. Consequently, successful design must consider both the enforcement of technological functions in real-world environments and whether users possess the corresponding cognitive capacity, skills, and social support to respond to these functions.

Second, the Theory of Media Affordances provides a systematic analytical framework for explaining how technological characteristics shape disease management practices. The aim of this study is to elucidate how the different affordances of DHTs influence the disease management practices and experiences of young adults with CVD. The three core dimensions of the Theory of Media Affordances (34) enable the systematic analysis of how specific patient experiences are enabled or hindered by the underlying functional properties of the technology, moving beyond superficial descriptions of fragmented experiences and thereby achieving the core research objective of in-depth explanation. As revealed by Schrock (60) in media technology research, the affordance perspective has helped uncover the profound restructuring effects of technology on social practices and daily routines.

Finally, this theory is particularly well-suited for analyzing the young adult patient population. This demographic is typically accustomed to deep, interactive engagement with technology, rather than passively receiving information (44). Concurrently, research has indicated that social support at the community level plays a positive role in improving patient rehabilitation adherence and clinical outcomes (57). Therefore, analyzing this group's interaction with DHTs through the lens of the Theory of Media Affordances not only aligns with their technology use habits but also reveals potential pathways for integration technology into health management from social and behavioral perspectives.

The findings of this study may provide valuable references for multiple stakeholders. Theoretically, the study can reveal the application value and boundaries of the Theory of Media Affordances within the health context, pointing to directions for future research. Practically, the synthesized evidence may offer user-centered design blueprints for healthcare practitioners and digital health technology developers, guiding the creation of support tools that better address the insidious and long-term management needs of young adults with CVD. Ultimately, by systematically integrating user experience evidence, this study aims to contribute to enhancing the suitability and effectiveness of digital health technologies targeted at young adults with CVD.

### Strengths and limitations

This study has several limitations. First, the field of DHTs is evolving rapidly, and the available evidence may not fully represent the latest technological applications and user experiences. Second, the heterogeneity of DHT types (e.g., wearable devices, mobile applications, telehealth platforms) may pose challenges for the integration and comparability of findings across studies. Third, variability in qualitative methodologies across primary studies may influence the quality of data and the feasibility of synthesis. Fourth, the inclusion of only English-language literature may introduce language bias, potentially limiting the generalizability of the findings across diverse cultural contexts. Finally, although the Theory of Media Affordances provides a valuable analytical perspective, it may also, to some extent, constrain other potential interpretive dimensions of the research findings.
