# Supplementary material for: Experiences of young adults with cardiovascular disease in using digital health: a protocol for a qualitative systematic review and meta-synthesis from the perspective of media affordances
Source: Front Cardiovasc Med. 2026 May 22;13:1806112. doi: 10.3389/fcvm.2026.1806112 (PMC13236932; doi:10.3389/fcvm.2026.1806112)
Supplement: Supplementary file 1 [file Datasheet1.docx]

Appendix 1

Pubmed search strategy

| **Search ID** | **Search Terms** |
| --- | --- |
| #1 | "digital health"[MeSH Terms] OR "digital technology"[MeSH Terms] OR "telemedicine"[MeSH Terms] OR "Digital Media"[MeSH Terms] OR "Information Technology"[MeSH Terms] OR "Digital Health Technolog*"[Title/Abstract] OR "digital technolog*"[Title/Abstract] OR "Digital Electronic*"[Title/Abstract] OR "mobile health"[Title/Abstract] OR "mHealth"[Title/Abstract] OR "Telehealth"[Title/Abstract] OR "eHealth"[Title/Abstract] OR "Telecare"[Title/Abstract] OR "Virtual Medicine"[Title/Abstract] OR "digital treatment*"[Title/Abstract] OR "e-health"[Title/Abstract] OR "m-health"[Title/Abstract] OR "online"[Title/Abstract] OR "mobile technolog*"[Title/Abstract] OR "Electronic Media"[Title/Abstract] OR "Information Technolog*"[Title/Abstract] |
| #2 | "wearable electronic devices"[MeSH Terms] OR "Smartphone"[MeSH Terms] OR "Telephone"[MeSH Terms] OR "Cell Phone"[MeSH Terms] OR "Wearable Electronic Device*"[Title/Abstract] OR "Wearable Device*"[Title/Abstract] OR "Wearable Technolog*"[Title/Abstract] OR "Electronic Skin"[Title/Abstract] OR "Wearable Computer*"[Title/Abstract] OR "Wearable"[Title/Abstract] OR "Smartphone*"[Title/Abstract] OR "Smart Phone*"[Title/Abstract] OR "Telephone*"[Title/Abstract] OR "Cellular Phone*"[Title/Abstract] OR "Cellular Telephone*"[Title/Abstract] OR "Cell Phone*"[Title/Abstract] OR "Mobile Phone*"[Title/Abstract] OR "Mobile Telephone*"[Title/Abstract] OR "Phone*"[Title/Abstract] OR "smartwatch*"[Title/Abstract] OR "smart watch*"[Title/Abstract] |
| #3 | "Text Messaging"[MeSH Terms] OR "Remote Patient Monitoring"[MeSH Terms] OR "mobile applications"[MeSH Terms] OR "Internet"[MeSH Terms] OR "Software"[MeSH Terms] OR "Texting*"[Title/Abstract] OR "Short Message Service"[Title/Abstract] OR "Text Message*"[Title/Abstract] OR "Mobile Application"[Title/Abstract] OR "Mobile App*"[Title/Abstract] OR "Portable Electronic App*"[Title/Abstract] OR "Portable Electronic Application*"[Title/Abstract] OR "Portable Software App*"[Title/Abstract] OR "Portable Software Application*"[Title/Abstract] OR "Smartphone App*"[Title/Abstract] OR "application*"[Title/Abstract] OR "App"[Title/Abstract] OR "software*"[Title/Abstract] OR "health app*"[Title/Abstract] OR "World Wide Web"[Title/Abstract] OR "Cyberspace"[Title/Abstract] OR "Cyber Space"[Title/Abstract] OR "Web"[Title/Abstract] OR "website"[Title/Abstract] OR "digital platform"[Title/Abstract] OR "online platform"[Title/Abstract] OR "Mobile platform"[Title/Abstract] |
| #4 | "social media"[MeSH Terms] OR "Social Networking"[MeSH Terms] OR "Social Medium"[Title/Abstract] OR "Social Media Messaging*"[Title/Abstract] OR "Mobile Social Media*"[Title/Abstract] OR "Mobile Social Network*"[Title/Abstract] OR "Social Network*"[Title/Abstract] OR "Facebook"[Title/Abstract] OR "Instagram"[Title/Abstract] OR "WhatsApp"[Title/Abstract] OR "Twitter"[Title/Abstract] OR "Tiktok"[Title/Abstract] OR "WeChat"[Title/Abstract] OR "Myspace"[Title/Abstract] OR "QQ"[Title/Abstract] OR "digital twins"[Title/Abstract] OR "virtual twin"[Title/Abstract] OR "cyber-physical system"[Title/Abstract] OR "computational model"[Title/Abstract] |
| #5 | "Artificial Intelligence"[MeSH Terms] OR "Computer Reasoning"[Title/Abstract] OR "Machine Intelligence"[Title/Abstract] OR "Computational Intelligence"[Title/Abstract] OR "Computer Vision System*"[Title/Abstract] OR "Knowledge Acquisition"[Title/Abstract] OR "Knowledge Representation*"[Title/Abstract] |
| #6 | #1 OR #2 OR #3 OR #4 OR #5 |
| #7 | "young adult"[MeSH Terms] OR "emerging adult*"[Title/Abstract] OR "youth*"[Title/Abstract] |
| #8 | "cardiovascular diseases"[MeSH Terms] OR "heart diseases"[MeSH Terms] OR "hypertension"[MeSH Terms] OR "heart failure"[MeSH Terms] OR "myocardial infarction"[MeSH Terms] OR "coronary artery disease"[MeSH Terms] OR "cardiomyopathies"[MeSH Terms] OR "cardiac event*"[Title/Abstract] OR "cardiac disorder*"[Title/Abstract] OR "high blood pressure"[Title/Abstract] OR "cardiac failure"[Title/Abstract] OR "heart attack"[Title/Abstract] OR "Arrhythmia"[Title/Abstract] OR "Dysrhythmia"[Title/Abstract] OR "coronary atheroscleros*"[Title/Abstract] OR "heart defect*"[Title/Abstract] OR "myocardial disease"[Title/Abstract] |
| #9 | "grounded theory"[MeSH Terms] OR "interviews as topic"[MeSH Terms] OR "focus groups"[MeSH Terms] OR "qualitative research"[MeSH Terms] OR "mix method*"[Title/Abstract] OR "case study"[Title/Abstract] OR "grounded theory"[Title/Abstract] OR "narrat*"[Title/Abstract] OR "field study"[Title/Abstract] OR "content analysis"[Title/Abstract] OR "thematic analysis"[Title/Abstract] OR "phenomen*"[Title/Abstract] OR "qualitative study"[Title/Abstract] OR "interview*"[Title/Abstract] |
| #10 | "experienc*"[Title/Abstract] OR "satisfact*"[Title/Abstract] OR "attitude*"[Title/Abstract] OR "opinion*"[Title/Abstract] OR "percep*"[Title/Abstract] OR "process*"[Title/Abstract] OR "feel*"[Title/Abstract] OR "view*"[Title/Abstract] OR "stor*"[Title/Abstract] OR "emotion*"[Title/Abstract] |
| #11 | ("2013/1/1"[Date - Publication] : "2025/8/31"[Date - Publication]) AND #6 AND #7 AND #8 AND #9 AND #10 |
